# Developmental Sialylation in Neuroblastoma and Neural-Crest-Derived Tumors

**DOI:** 10.3390/cancers18183023

**Published:** 2026-09-17

**Authors:** Laxmi Swami, Megha Sharma, Pracheta Janmeda, Punarvi Mandadapu, Fauzia Jamal, Pradeep Kumar, Karin M. Hardiman, Laura L. Stafman

**Affiliations:** 1 Department of Surgery, University of Alabama at Birmingham, Birmingham, AL 35233, USA; lswami@uabmc.edu (L.S.);; 2 Department of Bioscience and Biotechnology, Banasthali Vidyapith, Banasthali 304022, India; 3 Department of Cell, Developmental, and Integrative Biology, University of Alabama at Birmingham, Birmingham, AL 35233, USA; 4 Department of Radiology, University of Alabama at Birmingham, Birmingham, AL 35233, USA; pradeepkumar@uabmc.edu

**Keywords:** sialic acid, sialylation, neural crest, neuroblastoma, GD2, polysialic acid, ST8SIA1, glycocalyx, Siglec, developmental plasticity

## Abstract

Pediatric malignancies are often closely linked to developmental cell states, differentiation trajectories, and restricted developmental windows. This relationship is especially evident in neural-crest-derived tumors, where malignant cells may retain, remodel, or reacquire glycosylation programs that normally regulate embryonic migration and lineage maturation. Using a pediatric developmental perspective, we consider tumor-associated sialylation as potentially reflecting retention of the developmental cell-of-origin glycome, reacquisition during malignant cell state plasticity, or cancer-specific remodeling. Neuroblastoma provides the strongest evidence linking these mechanisms to tumor biology and therapy. Polysialylated NCAM can support migratory and plastic phenotypes, ganglioside biosynthesis is coupled to adrenergic-mesenchymal cell state through cell state-dependent regulation of glycosyltransferases including ST8SIA1, and the developmentally enriched ganglioside GD2 creates an actionable therapeutic vulnerability. Selected neural-crest-derived tumors that also occur outside childhood are included as they may help distinguish lineage-associated mechanisms from features specific to pediatric malignancy. We examine sialoglycan-Siglec immune regulation, emerging glycan-directed therapies, and approaches for resolving sialylation across developmental and malignant cell states. This framework argues that pediatric neural-crest-derived tumors do not simply recapitulate embryonic glycosylation but selectively retain, remodel, or reacquire developmental sialylation programs that can influence plasticity, migration, immune evasion, and therapeutic response.

## Simple Summary

Pediatric cancers frequently arise in the setting of disrupted differentiation and the persistence of developmentally immature cell states. Neural-crest-derived tumors provide an informative setting in which to ask whether malignant cells retain, remodel, or reacquire embryonic cell surface glycosylation programs. This review focuses on sialylation, a dynamic post-translational modification that regulates processes important in both development and cancer: adhesion, migration, signaling, membrane organization, and immune recognition. Developmental ganglioside pathways generate the therapeutic antigen GD2, polysialylated NCAM supports cellular plasticity and migration, and cell state transitions alter ST8SIA1 and GD2 abundance. Neuroblastoma is the clearest neural-crest-derived pediatric malignancy in which these pathways are altered, whereas melanoma and other neural-crest-derived tumors provide comparative systems for evaluating related mechanisms. Distinguishing developmental retention from tumor-acquired sialylation may reveal biomarkers and therapeutic vulnerabilities that are especially relevant to childhood cancer.

## 1. Introduction

The neural crest is a unique, multipotent group of cells that arise at the dorsal margin of the neural tube early in embryogenesis [1,2,3]. After undergoing an epithelial-to-mesenchymal transition (EMT), neural crest cells delaminate, migrate throughout the embryo, and differentiate into a broad range of tissue types including peripheral and enteric neurons and glia, Schwann cells, melanocytes, craniofacial bones and cartilage, and chromaffin cells in the adrenal medulla [4]. Because neural crest cells combine transient multipotency with migration and lineage diversification, they have long served as a model for studying developmental plasticity and cell-fate decisions. Many of these processes are enacted at the cell surface where glycosylation, and sialylation in particular, shapes the development of neural crest cells.

Sialylation is a comparatively underexplored component of this cell-surface regulatory layer, yet it shapes several of the developmental behaviors described above. Sialic acids are negatively charged terminal sugars on cell-surface and secreted glycoproteins and glycolipids. Their regulated addition and removal shape adhesion, receptor signaling, glycocalyx organization, and immune interactions [5]. During neural crest development, the strongest direct evidence concerns polysialylated neural cell adhesion molecule (polySia-NCAM), which reduces adhesive interactions and supports migration, while broader roles for sialylation in induction and lineage maturation remain less completely defined [6,7]. Several sialylated structures and their biosynthetic enzymes are also altered in neural-crest-derived tumors [6,8]. In neuroblastoma, for example, polySia-NCAM is linked to the developmental functions of polysialic acid in cell migration, and experimental disruption of polysialylation reduced neuroblastoma cell migration [9]. Clinically, polySia-NCAM expression in primary neuroblastomas has been associated with advanced or metastatic disease at diagnosis [10]. However, its relation to outcome is not unidirectional. In a tissue microarray cohort, absence of polySia-NCAM among patients with advanced disease was associated with markedly poorer survival [10]. These observations raise the possibility that malignant cells may selectively retain, reacquire, or remodel components of developmental sialylation programs rather than simply displaying nonspecific cancer-associated hypersialylation. These neuroblastoma-specific observations raise the broader questions of to what extent related sialylation programs operate across the wider family of neural-crest-derived tumors and what this reveals about the pediatric, developmental origins of the disease.

Several pediatric and young-adult tumors arise from, or retain developmental relationships to, neural-crest-derived lineages including neuroblastoma, melanoma, malignant peripheral nerve sheath tumors (MPNST), pheochromocytoma/paraganglioma, and schwannoma [11]. The developmental behaviors that characterize neural crest cells—EMT, adhesion remodeling, migration, survival away from the site of origin, and lineage plasticity—also resemble processes exploited during tumor invasion, metastasis, and therapy resistance [12,13,14].

Neuroblastoma, a pediatric malignancy of the developing sympathetic nervous system that is the most common extracranial solid tumor of childhood, provides the clearest example of this relationship [4,13]. Single-cell and developmental studies place neuroblastoma cells along sympathoadrenal trajectories and support a model in which malignant cells occupy or reacquire incompletely differentiated states rather than completing normal maturation [4,13,15]. Related developmental programs have been implicated in other neural-crest-derived tumors, including melanoma, MPNST, pheochromocytoma/paraganglioma, and schwannoma, although the strength and nature of the evidence differ substantially among tumor types [16,17,18,19,20].

The pediatric context is biologically important because many childhood cancers arise during periods of active tissue development and retain transcriptional features of incompletely differentiated progenitors. In this setting, a tumor-associated sialoglycan need not represent a program that has been reactivated after years of tissue maturation. Instead, it may reflect retention of a developmental glycan state, remodeling of that state as malignant cells move between developmental identities, or de novo acquisition of a glycan program that converges on developmental functions such as motility, plasticity, signaling, or immune regulation. These possibilities are not mutually exclusive. We therefore use a retention–reacquisition–remodeling framework throughout this review and treat neuroblastoma as the primary pediatric model in which developmental lineage, cell state, sialylation, and therapeutic antigen expression can be examined together.

**Figure 1:**
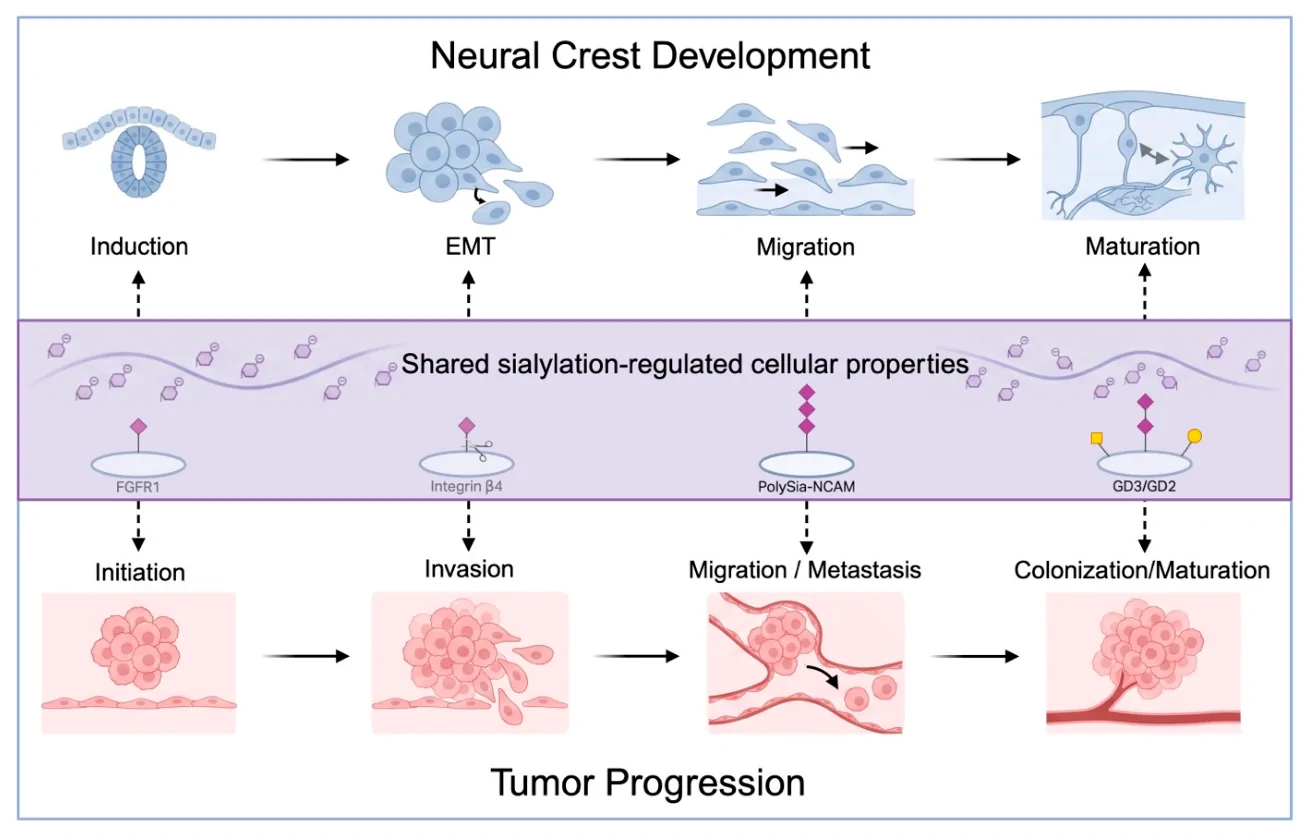
Conceptual parallels between neural crest development and neural-crest-derived tumor progression linked by shared sialylation-regulated cellular properties. Neural crest development (top) proceeds through induction at the neural plate border, epithelial-to-mesenchymal transition (EMT), migration along extracellular matrix-rich pathways, and maturation into differentiated peripheral lineages. Neural-crest-derived tumors (bottom) can exhibit conceptually analogous cellular behaviors, including tumor initiation, invasion, migration/metastasis, and colonization/maturation at distant sites. Sialylation (center band) can regulate cellular properties shared across these processes, including adhesion, receptor signaling, membrane organization, and immune recognition. Representative molecular examples discussed throughout this review are shown within this center band at each stage and differ in the strength of supporting evidence, from hypothesized or inferred from non-neural crest systems (FGFR1, integrin β4) to directly demonstrated in neural crest or neuroblastoma models (polySia-NCAM, GD3/GD2). The alignment between development and tumor progression is conceptual and does not imply stage-for-stage mechanistic equivalence. Figure created in BioRender. Stafman. (2026) https://app.biorender.com.

This review integrates developmental glycobiology with pediatric tumor biology and emphasizes the strength and type of evidence supporting each proposed developmental-tumor parallel (Figure 1). Throughout, we distinguish developmental or lineage evidence from human tumor association, functional or causal evidence, and clinical relevance. We use three non-exclusive interpretations for tumor-associated sialylation: (1) retention of a developmental glycan state, (2) reacquisition of a state that was lost during differentiation, and (3) de novo remodeling that converges on developmental functions. Where tumor-specific evidence is absent, developmental analogy is treated as a hypothesis rather than evidence of pathway activity in the tumor.

## 2. Fundamentals of Sialic Acid Biology

### 2.1. Structure and Types of Sialic Acids

Sialic acids are a family of acidic nine-carbon sugar molecules that commonly occupy terminal positions on glycans of cell-surface and secreted glycoproteins and glycolipids [5,7]. N-acetylneuraminic acid (Neu5Ac) is the most abundant and best-characterized sialic acid in humans. Other naturally occurring forms include 2-keto-3-deoxy-D-glycero-D-galactononic acid (KDN) [5,7]. Their terminal position makes sialic acids important determinants of molecular recognition at the cell surface. Their functional effect depends on the linkage. α2,3- and α2,6-linked sialic acids generate distinct glycan epitopes, whereas α2,8-linked sialic acids can form oligo- or polysialic acid chains that are especially relevant in neural biology [21,22]. These linkages are not interchangeable; each creates a structurally distinct epitope that acts like a precise key, determining which lectins, receptors, and sialic-acid-binding immunoglobulin-like lectins (Siglecs) on other cells can recognize and engage the cell surface.

### 2.2. Sialic Acid Biosynthesis

Sialic acids are generated through two convergent routes: (1) de novo synthesis from cytosolic UDP-GlcNAc and (2) a salvage pathway that recycles free sialic acid [5]. In de novo synthesis, UDP-GlcNAc is converted to Neu5Ac. Neu5Ac is then activated in the nucleus by CMP-sialic acid synthetase to form CMP-Neu5Ac. CMP-Neu5Ac is transported into the Golgi, where it serves as the donor substrate for sialyltransferases. The de novo pathway is tightly self-regulated by feedback inhibition, allowing cells to titrate sialylation to cellular demand [23]. Mechanistically, this feedback acts at the first committed step of the pathway when UDP-GlcNAc 2-epimerase/ManNAc kinase (GNE) is inhibited by CMP-Neu5Ac. Accumulation of the activated sugar donor throttles further flux into sialic acid biosynthesis and keeps cell-surface sialylation within a physiologic range.

### 2.3. Sialyltransferases

Sialyltransferases transfer sialic acid from CMP-Neu5Ac to glycoprotein and glycolipid acceptors. The 20 human sialyltransferases are grouped into ST3GAL, ST6GAL, ST6GALNAC, and ST8SIA families according to linkage and substrate preference [24]. Because acceptor preference is dictated by both sialyltransferase family and the specific N- or O-glycan structure presented by a given glycoprotein, any protein bearing the corresponding terminal galactose, GalNAc, or sialic acid residues is a potential substrate.

Individual enzymes are expressed in distinct tissue- and developmental stage-specific patterns, allowing the cell-surface glycome to change during development and disease [6,21]. This substrate logic underlies why glycoproteins known to carry N- or O-glycans with these terminal structures, including Wnt ligands and other secreted signaling proteins discussed below, represent plausible targets for sialyltransferase-mediated regulation.

### 2.4. Neuraminidases

Neuraminidases (sialidases) counterbalance sialyltransferases by removing terminal sialic acids from glycoconjugates. Mammals express four neuraminidases, NEU1–NEU4, with distinct substrate preferences and subcellular localization [22,25]. Sialyltransferases and neuraminidases are major determinants of local sialylation, but the resulting cell surface sialome also depends on precursor supply, CMP-Neu5Ac transport, acceptor abundance, competing glycosyltransferase pathways, trafficking, and glycoconjugate turnover. Together, these processes influence receptor availability, signaling, membrane organization, and cell surface charge.

### 2.5. Biological Functions Relevant to Development and Cancer

**Figure 2:**
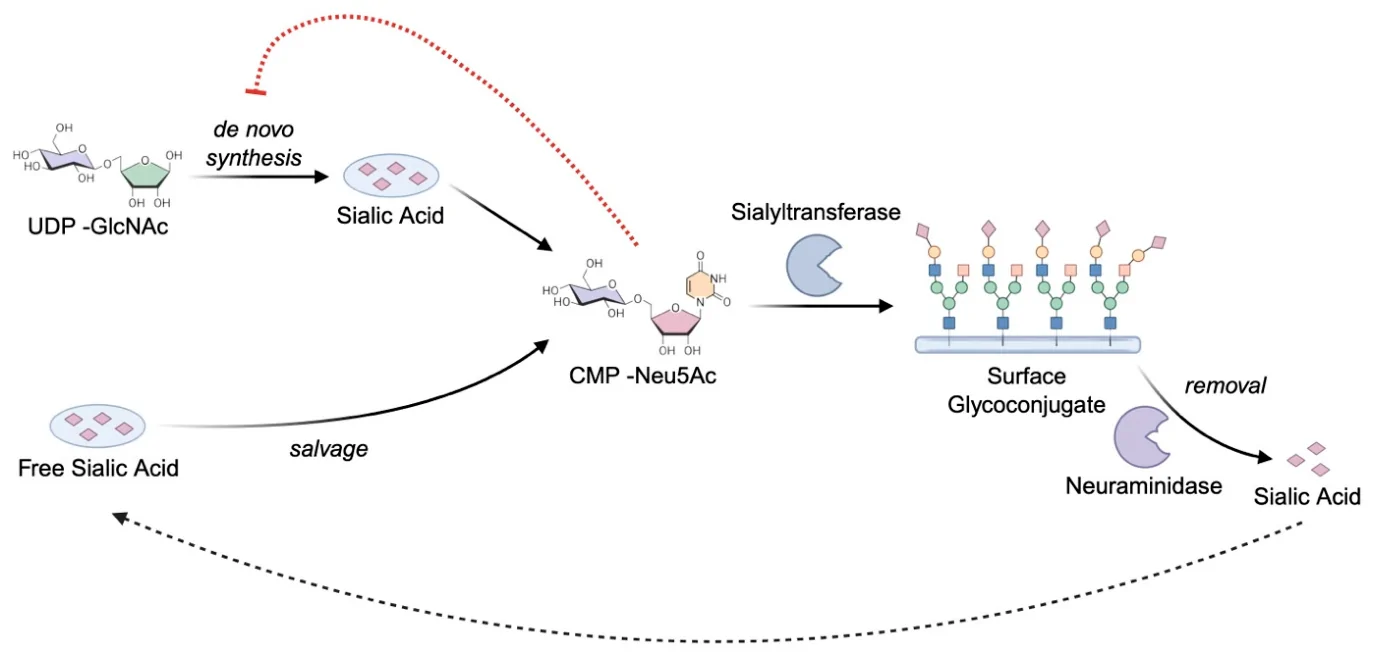
Sialic acid biosynthesis, activation, and the sialyltransferase–neuraminidase balance that sets cell surface sialylation. Sialic acids are generated through two convergent routes: de novo synthesis from cytosolic UDP-GlcNAc, and a salvage pathway that recycles free sialic acid. Both routes feed into CMP-Neu5Ac, the activated donor substrate produced by CMP-sialic acid synthetase. Negative feedback occurs in the de novo synthesis pathway when CMP-Neu5Ac inhibits the GNE enzyme. Following transport into the Golgi, sialyltransferases transfer sialic acid onto glycoprotein and glycolipid acceptors, whereas neuraminidases remove terminal sialic acids from glycoconjugates. These enzymes act within a broader network that includes precursor availability, acceptor abundance, competing glycosyltransferases, trafficking, and turnover. Together, these processes determine the sialylation of a cell surface glycoconjugate. Figure created in BioRender. Stafman. (2026) https://app.biorender.com.

Sialylation influences development and cancer through several overlapping mechanisms. Polysialylation can create substantial steric repulsion between neighboring cells. In neural development, this repulsion from polySia-NCAM reduces adhesive interactions and facilitates cell migration [7,8]. Sialylation also modifies receptor and integrin trafficking, ligand-binding affinity, and receptor clustering [5], while the size and charge of cell-surface glycans contribute to the mechanical properties of the glycocalyx, influencing how cells perceive and respond to mechanical cues from their surroundings [26]. Finally, sialoglycans can engage inhibitory Siglec receptors on immune cells, suppressing antitumor responses [27,28,29]. These functions—adhesion control, signaling, mechanics, and immune recognition—provide the mechanistic framework for the developmental and oncologic observations discussed below (Figure 2).

## 3. Sialylation in Neural Crest Development

Neural crest development proceeds through specification at the neural plate border, EMT and delamination, directed migration, and differentiation into diverse peripheral lineages [30]. Sialylation changes across this sequence, but the evidence is not equally strong at every developmental stage. The best-established neural-crest-related function involves adhesion and migration through polySia-NCAM. By contrast, proposed roles in induction, EMT, and lineage specification rely more heavily on broader glycan biology and developmental model systems. This distinction is important because the presence of sialylation pathways in a neural-crest-derived tumor does not, by itself, demonstrate reuse of a neural crest developmental program.

### 3.1. Neural Crest Induction and Developmental Signaling

Neural crest identity is established by coordinated Wnt, BMP, and FGF signaling and a conserved gene-regulatory network that includes neural plate border factors such as PAX3/7, ZIC1, and MSX1/2 and neural crest specifiers including SNAI2, FOXD3, and SOX9/10 [3,30]. Glycans clearly participate in this signaling environment. Heparan sulfate proteoglycans regulate the availability and spatial range of BMP ligands and antagonists at the neural plate border [31].

Direct evidence that sialylation itself controls neural crest induction is limited, but sialylation of individual components in these pathways has been demonstrated in other developmental and cellular contexts. FGFR1 is a direct substrate of ST6GAL1-mediated α2,6-sialylation, which potentiates downstream ERK and FAK signaling [32]. Polysialic acid carried on NCAM sequesters FGF2 and delivers it to heparan sulfate and the FGF receptor [33]. Wnt and BMP pathway components are also glycoproteins. Wnt-3a secretion and Frizzled4/LRP6 trafficking depend on N-glycosylation, and BMPR2 N-glycosylation enhances ligand binding [34,35,36,37], but whether these N-glycans are capped with sialic acid and whether that sialylation is functionally required have not been established. To our knowledge, no lectin-precipitation or site-specific glycoproteomic study has directly tested whether Wnt-3a, Frizzled4, LRP6, or BMPR2 carry terminal sialic acid in neural crest or other embryonic stem cell models. This remains an experimental gap, and closing this gap will require glycan-selective lectin pulldown or mass spectrometry-based glycoproteomics applied directly to these receptors during neural crest induction.

Because sialylation can alter receptor presentation, clustering, and trafficking in other developmental contexts [6], it is a plausible regulator of inductive signaling thresholds, but this remains a hypothesis rather than an established neural crest mechanism.

### 3.2. Epithelial-to-Mesenchymal Transition (EMT)

Neural crest delamination requires extensive remodeling of epithelial adhesion programs [14,38]. In non-neural crest epithelial models, TGF-β-induced EMT is accompanied by dynamic changes in cell-surface sialylation, and pharmacologic inhibition of sialylation can accelerate EMT, in part through desialylation of integrin β4 [39]. Human pluripotent stem cell-derived neural crest likewise shows marked remodeling of glycan epitopes and integrins during EMT, including loss of neuroepithelial glycan markers (CD15, CD133, CD49f, CD57/HNK1) and a concurrent rise in integrin markers (CD49d, CD49e, and CD51/CD61) [40]. These observations establish glycan remodeling as a feature of neural crest EMT, but they do not yet demonstrate that a specific sialylation change is required for delamination.

### 3.3. Neural Crest Migration

After delamination, neural crest cells migrate along extracellular-matrix-rich pathways that prominently include laminin and fibronectin [41]. Here the evidence for a direct sialylation mechanism is stronger. PolySia is added to NCAM in a developmentally regulated manner and increases intermembrane spacing, thereby weakening both homophilic and heterophilic NCAM–NCAM and NCAM–matrix interactions [42,43,44]. In enteric neural crest precursors, BMP4 regulates the extent of NCAM polysialylation and thereby alters adhesive behavior during gangliogenesis [45]. PolySia-NCAM therefore provides a clear example in which regulated sialylation directly controls a property, adhesion, that is essential for neural crest migration.

### 3.4. Differentiation of Neural Crest Derivatives

As neural-crest-derived and related neural progenitors mature, polysialylation generally declines and ganglioside composition becomes more complex [44,46]. In embryonic chick nervous tissue, GD3 is abundant in proliferating neuroepithelial and migrating neural crest cells and decreases with differentiation [47]. In the postnatal mouse brain, loss of GD3 synthase depletes neural stem cells and impairs neurogenesis, supporting a role for GD3 in maintenance of progenitor states [48], although this is not a neural-crest-specific finding. Thus, the developmental data support a broad transition from migratory, proliferative states toward differentiated glycan states, but not a single universal neural crest sialylation program.

The central question for cancer biology is therefore whether tumors selectively retain or reacquire sialylation states that confer developmental properties such as plasticity, motility, or altered signaling.

## 4. Developmental Reprogramming of Sialylation in Pediatric Neural Crest-Derived Tumors

Elements of the developmentally regulated sialylation reappear or are remodeled after malignant transformation. The strongest evidence comes from neuroblastoma, where cell state, ganglioside synthesis, polysialylation, and sympathoadrenal differentiation intersect. Evidence across other neural-crest-derived tumors is more uneven. Where direct data exist, altered sialyltransferases, neuraminidases, and sialoglycan structures can influence tumor growth, migration, immune interactions, and therapeutic sensitivity.

### 4.1. Pediatric Tumors as Disorders of Developmental Arrest and Lineage Plasticity

Neuroblastoma is increasingly viewed through a developmental framework in which malignant cells occupy incompletely differentiated sympathoadrenal states [49]. Tumors contain two major, interconvertible transcriptional identities: an adrenergic (ADRN) state resembling committed sympathoblasts, and a mesenchymal (MES) state with neural-crest-like, EMT-associated, and Notch-associated features [50,51]. Transitions between these states occur spontaneously and under therapeutic pressure and contribute to intratumoral heterogeneity and treatment resistance [52]. For sialic acid biology, this plasticity is especially important because the ADRN-MES axis is accompanied by remodeling of the sialylation machinery rather than preservation of a fixed embryonic glycan state.

Developmental dysregulation is a broader theme in pediatric oncology. Wilms tumor, rhabdomyosarcoma, and medulloblastoma each retain transcriptional features of incompletely matured renal, myogenic, or neural progenitors, respectively [53,54,55]. These examples provide context for neuroblastoma but do not establish a shared sialylation mechanism across pediatric cancers. The remainder of this review therefore focuses on evidence specific to neural-crest-derived tumors and distinguishes cell state associations from direct glycan mechanisms.

### 4.2. Aberrant Sialyltransferase Expression

Tumor cell state transitions are accompanied by selective remodeling of sialylation machinery. In neuroblastoma, ST8SIA1 (GD3 synthase) is enriched in ADRN cells and sharply downregulated in MES cells. Forced expression of MES-associated transcription factors such as PRRX1 or NOTCH3 suppresses ST8SIA1, creating a bottleneck in GD2 synthesis [56]. The available evidence supports chromatin-mediated repression involving H3K27me3/PRC2-EZH2. The *ST8SIA1* promoter shows increased trimethylation of H3K27me3 in GD2-low mesenchymal cell lines, and pharmacologic inhibition of EZH2, the catalytic subunit of the H3K27-methylating Polycomb repressive complex 2 (PRC2), erases this mark and restores *ST8SIA1* transcription. A primary role for promoter DNA methylation has not been established. Yes-Associated Protein (YAP) provides an additional regulatory route. Genetic YAP inhibition in mesenchymal neuroblastoma increases ST8SIA1 and surface GD2, enhancing sensitivity to GD2-targeted immune killing [57]. ST8SIA2, a polysialyltransferase that contributes to polySia-NCAM synthesis, is broadly expressed across neuroblastoma and has been proposed as a molecular marker of metastatic or minimal residual disease [58]. Sialyltransferase dysregulation is not unique to neuroblastoma. In melanoma, ST3GAL1 and ST3GAL2 are upregulated relative to benign nevi and are required for melanoma growth through stabilization of the growth-signaling cell surface protein CD98hc [59], while ST3GAL1 also drives invasion and metastasis via sialylation-dependent dimerization and activation of AXL [60]. ST6GAL1, which adds α2,6-linked sialic acid, is upregulated in several cancers and linked to tumor-initiating and cancer stem cell phenotypes [61,62]. Collectively, these findings support tumor- and state-specific remodeling as opposed to a uniform increase in total sialylation.

### 4.3. Altered Glycocalyx

Dysregulated sialylation can reshape the glycocalyx in ways that influence adhesion and signaling [63]. PolySia-NCAM is the clearest developmental bridge. The same steric mechanism that weakens adhesion during development can facilitate motility when polySia is expressed by tumor cells [42,43]. Other sialylated structures, including selectin-binding glycans, contribute to metastatic adhesion in cancer more broadly, although these mechanisms have not been established uniformly in neural-crest-derived pediatric tumors [61,63,64]. In neuroblastoma, cell-state-dependent changes in ganglioside synthesis provide a particularly direct connection between lineage identity and cell surface glycosylation [56]. Thus, the tumor glycocalyx is best viewed as a dynamic readout and potential effector of cell state, rather than as a single pro-metastatic pathway.

## 5. Neuroblastoma: A Glycobiology Case Study

Neuroblastoma is the most common extracranial solid tumor of childhood and arises within the developing sympathetic nervous system [4,65]. Despite intensive multimodal therapy, survival for children with high-risk disease remains substantially worse than for most other pediatric cancers [65]. Among the sialic acid pathways discussed in this review, GD2 has the clearest clinical impact, making neuroblastoma the most informative model for linking developmental glycobiology with pediatric cancer.

### 5.1. GD2 Biology

GD2 is a disialoganglioside enriched during neural development and expressed at high density on most neuroblastomas. However, GD2 should not be treated as a simple binary tumor marker. Immunohistochemistry of 45 neuroblastomas recently demonstrated graded intratumoral heterogeneity, with 14 tumors (31%) containing < 90% GD2-positive tumor cells and spatial coexistence of GD2-positive and GD2-negative populations [66]. In postnatal tissues, GD2 expression is much more restricted, although it is detectable in the central nervous system, peripheral sensory nerves, melanocytes, and selected other cell types [67,68]. This differential expression creates a therapeutic window rather than absolute tumor specificity. Anti-GD2 antibodies therefore produce clinically meaningful antitumor activity but also predictable on-target toxicities, particularly a characteristic neuropathic pain syndrome [67]. The pain is due to off-target effects of anti-GD2 antibody binding GD2 on non-myelinating Schwann cells and nociceptive afferent fibers, triggering complement activation, membrane attack complex formation, and release of C5a, which sensitizes and depolarizes peripheral nociceptors, producing mechanical allodynia [69]. Demyelinating sensorimotor neuropathy has also been reported with some anti-GD2 antibodies due to direct antibody binding to peripheral nerve myelin [70].

GD2-directed monoclonal antibodies are now an established component of therapy for high-risk neuroblastoma and mediate antibody- and complement-dependent cytotoxicity [71]. Relapse nevertheless remains common, motivating a wave of second-generation GD2-directed strategies including chimeric antigen receptor (CAR) T cells, bispecific antibodies, radioimmunoconjugates, and combination regimens [71,72].

### 5.2. Gangliosides in Neuroblastoma

GD2 is synthesized from lactosylceramide (LacCer) through sequential activity of ST3GAL5 (GM3 synthase), ST8SIA1 (GD3 synthase), and B4GALNT1 (GM2/GD2 synthase) [73,74]. This pathway is developmentally regulated, and its intermediates are not merely passive membrane markers, as GD3 and GD2 can participate in membrane organization and signaling. Beyond ganglioside synthesis, these gangliosides participate in a range of biological processes, including neuronal differentiation and NF-κB- and CREB-dependent transcription, and GD3 itself associates directly with the epidermal growth factor receptor (EGFR) to sustain neural stem cell proliferation [74].

GD2 is concentrated in membrane lipid rafts, where gangliosides can influence the organization of signaling complexes. In neuroblastoma models, GD2-mediated signaling itself promotes tumor growth, migration, and proliferation through activation of Src kinase, phosphorylation of N-methyl-D-aspartate (NMDA) receptors, and stimulation of the FAK/AKT/ERK/mTOR signaling cascade [74]. These observations suggest that GD2 abundance can affect tumor cell behavior in addition to serving as a surface antigen, although the relative contribution of GD2 itself versus the broader ganglioside composition of lipid rafts remains context-dependent. Flux through the ganglioside pathway also affects GD2 abundance. Because B3GALT4 acts one step downstream of GD2 in the ganglioside pathway, converting it into more complex gangliosides, silencing B3GALT4 causes GD2 to accumulate rather than be consumed, thereby increasing lipid raft formation and downstream signaling activity. Conversely, restoring B3GALT4 expression lowers GD2 density, reduces lipid raft formation, and dampens this signaling in neuroblastoma models [46,75]. As GD2 is positioned between these two enzymes, its prevalence is determined by the balance between B4GALNT1-driven synthesis and B3GALT4-driven consumption, as opposed to either enzyme alone. B3GALT4 silencing shifts this balance toward net GD2 accumulation even without any change in B4GALNT1 activity itself.

Sialic acid remodeling in neuroblastoma extends beyond the canonical GD2 pathway. ST6GAL2, an α2,6-sialyltransferase is enriched in neural tissues and was first cloned from a neuroblastoma cell line, but its functional role in neuroblastoma remains largely unexplored [76]. Conversely, overexpression of the long neuraminidase isoform NEU4L in neuroblastoma cells activates β-catenin signaling, increases *MYCN* and other β-catenin targets and promotes a more proliferative, stem-like phenotype [77]. These observations illustrate both the breadth of sialic acid regulation in neuroblastoma and the large number of enzymes whose tumor-specific functions remain unresolved.

### 5.3. Polysialic Acid

Polysialylation is another developmentally regulated feature of neuroblastoma. PolySia-NCAM reduces cell–cell adhesion, and experimental increases in polysialylation enhance migration and invasion in neuroblastoma cell models [78]. Clinical associations are more complex. In a tissue microarray study, polySia-NCAM expression was associated with metastatic and advanced-stage disease, whereas among patients with advanced neuroblastoma, absence of polySia-NCAM-positive tumor cells was associated with poorer overall survival, particularly in tumors without *MYCN* amplification [10,78]. This apparent paradox argues against treating polySia-NCAM as a simple linear marker of aggressiveness and instead suggests that it may reflect tumor differentiation or cell state as well as migratory capacity. Because polysialylation of NCAM persists broadly across neuroblastoma tumors and cell lines, the polysialyltransferase responsible for this modification, ST8SIA2, has been proposed as a sensitive molecular marker for detecting minimal residual disease [58].

PolySia may additionally influence innate immune interactions. In vitro, low-molecular-weight polySia binds the complement regulatory protein properdin and blunts activation of the alternative complement pathway, protecting rat neuroblastoma cells from complement-mediated killing [79]. Whether this mechanism contributes materially to human neuroblastoma immune evasion in vivo remains to be established but raises a potential clinical tension, as complement-dependent cytotoxicity is one of the principal mechanisms of anti-GD2 antibodies. If tumor-associated polySia binds properdin and dampens the alternative complement pathway to a similar degree in vivo, it would be predicted to blunt rather than support anti-GD2 complement-dependent cytotoxicity. Whether local polySia density attenuates GD2 antibody-mediated complement-dependent cytotoxicity in patients has not been tested and represents an important question for reconciling this immune evasion mechanism with GD2-targeted therapies.

### 5.4. MYCN and Glycosylation

*MYCN* amplification is present in approximately 25% of neuroblastoma and is strongly associated with poor prognosis and aggressive disease [80]. *MYCN* drives neuroblastoma progression by blocking differentiation, sustaining proliferation, promoting genomic instability, accelerating angiogenesis, and facilitating immune evasion [80,81,82]. *MYCN* also plays a major role in metabolic reprogramming, including alterations in glycolysis, amino acid metabolism, polyamine biosynthesis, and glycosylation pathways [83]. Glycomic profiling of neuroblastoma cell lines demonstrated that *MYCN*-amplified cells were enriched for larger, more extensively sialylated glycans than their non-amplified counterparts [84]. More recently, these observations were extended to primary neuroblastoma tissues with the identification of distinct glycan profiles associated with *MYCN* amplification, including altered sialylation and fucosylation [85]. Together, these findings support an association between *MYCN* amplification and altered glycosylation.

**Figure 3:**
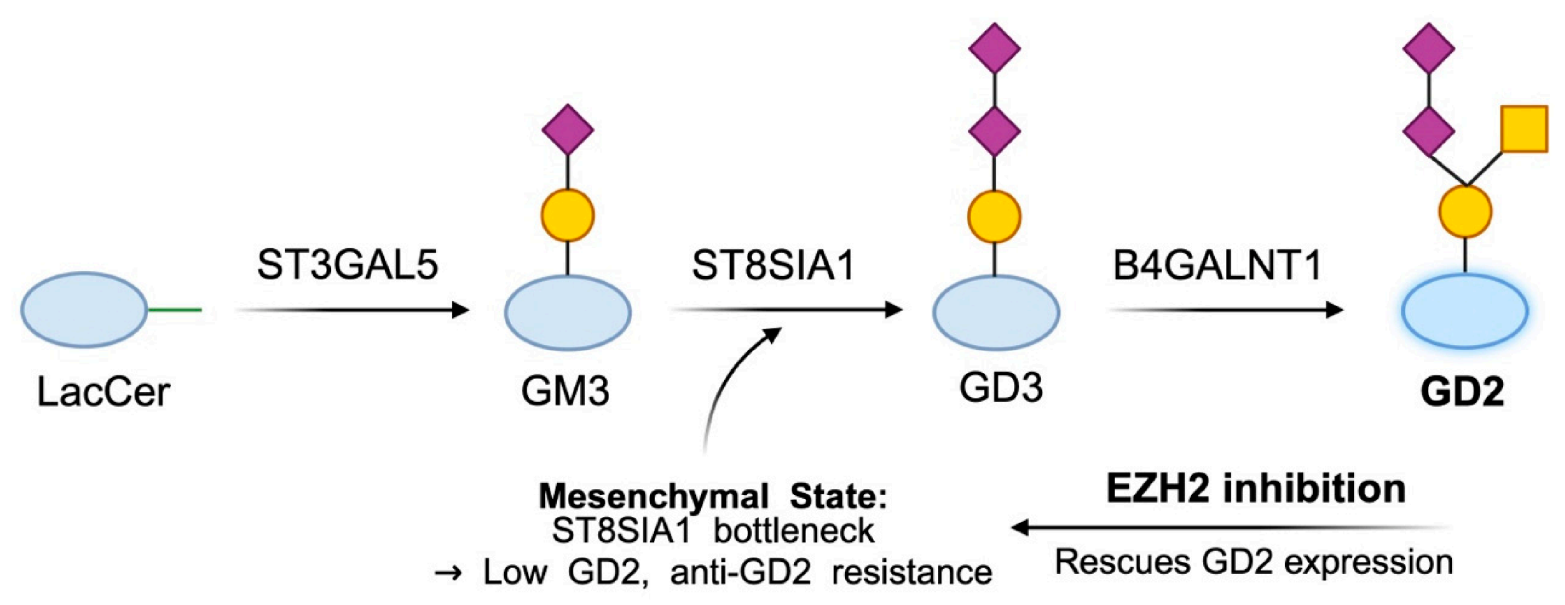
GD2 ganglioside biosynthesis and its state-dependent suppression in neuroblastoma. GD2 is synthesized from lactosylceramide (LacCer) through sequential activity of three enzymes: ST3GAL5 (GM3 synthase) generates GM3, ST8SIA1 (GD3 synthase) converts GM3 to GD3, and B4GALNT1 (GM2/GD2 synthase) converts GD3 to GD2. Because ST8SIA1 activity is a key bottleneck for GD2 synthesis, its regulation determines surface GD2 density. In the MES neuroblastoma cell state, ST8SIA1 expression is suppressed, lowering GD2 expression and conferring resistance to anti-GD2 immunotherapy. Because this suppression is epigenetically enforced, EZH2 inhibition can reprogram MES cells to restore ST8SIA1 expression and rescue GD2 expression, resensitizing tumor cells to GD2-targeted treatment. Figure created in BioRender. Stafman. (2026) https://app.biorender.com.

The most direct connection between neuroblastoma cell state and glycosylation is ADRN-MES control of GD2. MES neuroblastoma cells have reduced ST8SIA1, the GD3 synthase that gates GD2 synthesis, thereby lowering cell surface GD2 and conferring resistance to anti-GD2 immunotherapy. Because this silencing is epigenetically enforced, EZH2 inhibition can reprogram MES cells, restore ST8SIA1 and GD2 expression, and resensitize models to GD2-targeted treatment (Figure 3) [56]. YAP also converges on this bottleneck. YAP inhibition increases ST8SIA1 and GD2 and enhances immune killing by GD2-targeted therapies in mesenchymal models [57]. Human tumor data showing spatially intermixed GD2-high and GD2-low/negative populations further support GD2 density and heterogeneity as biologically relevant features [66]. These studies demonstrate that neuroblastoma cell state programs can directly alter a clinically actionable glycan antigen. Whether every component of this state-dependent program represents retention or reacquisition of a normal developmental glycan state requires direct comparison with matched human developmental lineages.

### 5.5. Tumor Microenvironment

The neuroblastoma tumor microenvironment contains diverse myeloid, lymphoid, endothelial, and stromal populations whose abundance and activation state vary with tumor differentiation and clinical context [86,87]. Neuroblastoma also employs multiple immune-evasion mechanisms, including altered antigen presentation and weak expression of some NK cell-activating ligands [86,87,88]. For the purposes of sialic acid biology, the most informative question is how tumor glycans modify these immune interactions.

GD2 itself participates in a glyco-immune checkpoint. In preclinical neuroblastoma models, tumor-cell GD2 binds the inhibitory immunoreceptor Siglec-7, which is expressed on macrophages and NK cells. Anti-GD2 antibodies disrupt the GD2–Siglec-7 interaction and increase surface expression of the pro-phagocytic signal calreticulin. Combined blockade of GD2 and CD47 consequently shifted macrophage activity toward phagocytosis and produced potent tumor clearance in neuroblastoma preclinical models [89]. These findings connect a tumor-associated ganglioside to both direct immunotherapeutic targeting and regulation of innate immune recognition. However, whether this glyco-immune checkpoint is strictly ganglioside-driven in neuroblastoma remains unknown. In other solid tumors, including pancreatic cancer, Siglec receptors on tumor-associated macrophages recognize sialylated glycoprotein, rather than glycolipid, ligands and drive macrophage reprogramming and immunosuppression [90,91]. This raises the possibility that neuroblastoma tumor-associated macrophages engage additional sialylated glycoproteins beyond GD2, but this has not yet been tested in neuroblastoma.

Clinical anti-GD2 therapy remains limited by pain and other toxicities, incomplete response, relapse, and antigen-low or antigen-negative cell states [71,72]. The lineage-dependent loss of ST8SIA1 and GD2 described above provides a mechanistic example of how developmental plasticity can undermine a glycan-targeted therapy [56].

## 6. Other Neural-Crest-Derived Tumors

Beyond neuroblastoma, the evidence for sialic acid biology is highly uneven across neural-crest-derived tumors (Table 1). Melanoma has substantial mechanistic evidence for cancer-associated sialylation, although a direct developmental neural crest link is not established for every pathway. MPNST, pheochromocytoma/paraganglioma, and schwannoma are supported mainly by developmental analogy, model system observations, or limited descriptive studies.

The primary emphasis of this review is neuroblastoma, the pediatric neural-crest-derived malignancy for which developmental sialylation evidence is most extensive. Other neural-crest-derived tumors, several of which can also occur outside childhood, are included as a comparative framework rather than as an equally weighted focus. They provide mechanistic evidence that helps distinguish pediatric developmental persistence from more general lineage-associated or cancer-associated sialylation. Melanoma is particularly informative in this regard because functional studies of sialyltransferases and gangliosides are more extensive than in several rarer pediatric neural crest tumors.

**Table 1:** Strength of evidence for sialylation pathways across neural-crest-derived tumor types. For each tumor type discussed in this review, the table lists the sialylation pathway or axis addressed, the strength of evidence supporting a role for that pathway in that specific tumor type, and the manuscript’s supporting citations. Established mechanism denotes functional evidence supporting causality in the specified tumor context. Correlative human data denotes associations demonstrated in patient specimens without establishing causality. Model-system only denotes evidence restricted to experimental models. Hypothesis/inferred denotes proposed relationships lacking direct evidence in that tumor type.

Tumor Type	Sialylation Pathway/Axis	Protein/Lipid Carrier(s)	Evidence Category	Basis for Category	Refs.
Neuroblastoma	GD3 synthase → GD2 ganglioside axis	GD3, GD2	Established mechanism	ST8SIA1 loss confers anti-GD2 resistance; EZH2 inhibition restores GD2; clinically actionable	[56,73,74]
Neuroblastoma	PolySia-NCAM	NCAM	Correlative human data	Experimental disruption of polysialylation reduces migration in cell models; clinically correlated with stage	[9,10,78]
Neuroblastoma	ST8SIA2	NCAM	Correlative human data	Broadly expressed across tumors/cell lines; proposed as a minimal residual disease marker	[58]
Neuroblastoma	*MYCN*-associated	Not carrier-specific	Correlative human data	Glycomic profiling associates *MYCN-amplified* tumors with more extensive sialylation; association only	[83,84,85]
Neuroblastoma	ST6GAL2	Not established	Model-system only	Cloned from cell line; no known functional role	[76]
Neuroblastoma	NEU4L	Not established	Model-system only	Overexpression in cell lines activates β-catenin signaling	[77]
Neuroblastoma	GD2-Siglec-7 checkpoint	GD2	Model-system only	Preclinical models only	[89]
Melanoma	ST3GAL1/ST3GAL2	CD98hc/SLC3A2	Established mechanism	Upregulated in melanoma vs. nevi; requirement for tumor growth via CD98hc stabilization and AXL activation	[59,60]
Melanoma	ST8SIA1-GD3 axis	GD3	Established mechanism	Increases progression and metastasis; poor survival; alters proliferation, invasion, lipid metabolism, tumor growth	[92,93]
Melanoma	ST6GAL1	Not established in melanoma	Model-system only	Upregulated in *BRAF*-mutant melanoma cell lines and identified in human *BRAF*-associated transcriptional signature	[94]
Melanoma	Siglec-sialoglycan interaction	Not established	Hypothesis/inferred	Inferred	[95]
MPNST	PolySia-NCAM	NCAM	Hypothesis/inferred	Inferred by analogy to peripheral nerve developmental biology	[96,97]
Pheochromocytoma/Paraganglioma	PolySia-NCAM	NCAM	Model-system only	Based on rat PC-12 pheochromocytoma cell line data	[98]
Schwannoma	PolySia-NCAM	NCAM/CD56	Correlative human data	Variation across human Schwann cell tumor and neuropathic tissue samples	[99]

### 6.1. Melanoma

Melanoma has the strongest non-neuroblastoma evidence for functional sialylation dysregulation. Early studies demonstrated stage-dependent changes in melanoma gangliosides. GD2 and the glycosyltransferase activity responsible for its synthesis from GD3 were detected in advanced primary and metastatic melanoma but not in normal melanocytes, suggesting that activation of the GD3-GD2 biosynthetic pathway accompanies acquisition of a more aggressive phenotype [100]. More recent studies have specifically implicated ST8SIA1 (GD3 synthase), which converts GM3 to GD3 and enables downstream GD2 synthesis. Ramos et al. found that ST8SIA1 and its product GD3 are increased during melanoma progression, with particularly high ST8SIA1 expression reported in melanoma brain metastases [92]. High ST8SIA1 expression was also associated with poorer overall survival in patients with lymph node metastases, while functional manipulation of ST8SIA1 altered melanoma cell proliferation and colony formation, supporting a role for the ST8SIA1-GD3 axis in melanoma progression. Recent work has strengthened the mechanistic evidence linking ST8SIA1 to melanoma aggressiveness. Liu et al. demonstrated that ST8SIA1 promoted melanoma proliferation, migration, invasion, tumor growth, and metastasis in gain- and loss-of-function models [93]. Proteomic and metabolomic analyses implicated lipid metabolic reprogramming as a downstream mechanism. Together, these findings provide human correlative and experimental evidence that remodeling of the ST8SIA1-GD3 ganglioside axis is associated with melanoma progression and metastatic behavior.

ST3GAL1 and ST3GAL2 are upregulated at both the transcriptional and protein levels in melanoma relative to benign nevi and are required for tumor proliferation [59]. Functional screens indicate that ST3GAL1- and ST3GAL2-mediated sialylation supports melanoma maintenance in part by stabilizing CD98hc [59]. ST3GAL1 is also induced by the SOX2-GLI1 transcriptional complex and promotes invasion and metastasis through AXL dimerization and activation [60].

Sialylation additionally intersects with melanoma’s oncogenic BRAF-MAPK signaling. ST6GAL1, which attaches an α2,6-linked sialic acid on N-glycans, is consistently upregulated in melanoma cell lines carrying activating BRAF mutations. *ST6GAL1* was identified as one of the few glycosylation genes within the BRAF-associated transcriptional signature, linking oncogenic RAS-BRAF-MAPK signaling directly to the sialylation machinery [94]. Finally, inhibitory Siglec–sialoglycan interactions are increasingly recognized as part of melanoma immune evasion, although the clinical utility of targeting this axis remains under investigation [95].

### 6.2. Malignant Peripheral Nerve Sheath Tumors

MPNST is an aggressive peripheral nerve sheath sarcoma that arises sporadically or in the setting of neurofibromatosis and is thought to emerge from Schwann cell precursors [101]. Tumor progression includes major lineage-stage and mesenchymal remodeling, with SOX9 identified as both a marker of this transition and a driver of tumor growth [102].

Direct evidence for sialylation in MPNST is minimal. In peripheral nerve development and regeneration, polySia is dynamically regulated by ST8SIA2 and ST8SIA4 and can influence axonal regeneration [96]. PolySia-NCAM is low in mature peripheral nerves, while re-expression has been linked to invasiveness in other tumors [97]. A plausible epigenetic route for such reactivation is suggested by recent work on PRC2. Loss-of-function mutations in PRC2 are recurrent in MPNST and cause genome-wide loss of the repressive H3K27me3 mark [103]. Chromatin profiling also shows that PRC2-deficient MPNST cells gain active enhancers at early neural crest regulators, driving a dedifferentiated, neural crest stem-cell-like transcriptional state that is not seen in PRC2-intact neurofibromas [104]. This provides a mechanistic parallel to the EZH2/H3K27me3-dependent regulation of *ST8SIA1* described for neuroblastoma in Section 4.2, but direct data in MPNST models have not yet been reported, and whether MPNSTs reactivate polySia-NCAM or other neural-crest-associated sialylation programs has not been established and represents a clear experimental gap.

### 6.3. Pheochromocytoma/Paraganglioma

Pheochromocytomas and paragangliomas are rare neuroendocrine tumors arising from adrenal chromaffin cells or extra-adrenal paraganglia, respectively [105]. Direct evidence for sialic acid regulation in human tumors is extremely limited. The principal mechanistic clue comes from rat pheochromocytoma-derived PC-12 cells, in which polySia-NCAM abundance and chain length change with growth stage and retinoic-acid-induced differentiation, mirroring the pattern described in neuroblastoma cells (Section 5.3) [98]. Thus, the available evidence supports differentiation-sensitive polysialylation in model systems, not a demonstrated sialylation program in human pheochromocytoma/paraganglioma.

Rather than relying solely on data from a rat cell line, it is worth considering human pheochromocytoma/paraganglioma within the framework of its dominant genetic and metabolic biology. A molecularly defined subset of these tumors harbor inactivating mutations in the von Hippel–Lindau gene (VHL) or in genes encoding succinate dehydrogenase (SDHx). These alterations produce pseudohypoxic signaling through impaired oxygen sensing or accumulation of the oncometabolite succinate, respectively, with consequent stabilization and activation of hypoxia-inducible factors HIF-1α and HIF-2α and broader metabolic and epigenetic reprogramming [106]. Whether pseudohypoxic signaling directly regulates sialyltransferase transcription or CMP-Neu5Ac generation in pheochromocytoma/paraganglioma specifically has not been studied. However, a mechanistic link between hypoxia signaling and sialylation exists in other tumor types. In ovarian and pancreatic cancer cells, ST6GAL1 enhances HIF-1α stabilization and signaling [107], establishing precedent for crosstalk between the HIF axis and sialylation. Testing whether an analogous relationship exists under the pseudohypoxic conditions of VHL- or SDH-mutant pheochromocytoma/paraganglioma represents a concrete opportunity to move beyond model-system inference toward a mechanism grounded in these tumors’ genetic drivers.

### 6.4. Schwannoma

Schwannomas are benign Schwann cell tumors that commonly express CD56/NCAM [108]. PolySia-NCAM expression has been reported to vary among Schwann cell tumors and neuropathic tissues [99]. Whether this heterogeneity has functional or prognostic significance in schwannoma is unknown. As in MPNST, the key next step is to pair tumor glycomic profiling with the well-described developmental biology of the Schwann cell lineage.

## 7. Therapeutic Targeting of Sialic Acid Biology

Sialic acid biology creates three broad therapeutic opportunities: targeting defined sialylated antigens, altering tumor sialylation itself, and interrupting sialoglycan–Siglec immune checkpoints. GD2-directed therapy in neuroblastoma is the clinical proof of principle, whereas most strategies aimed at the broader sialome remain predominantly preclinical or early-stage clinical.

### 7.1. Clinically Established Targeting of GD2

GD2 is the most clinically mature target in this field. Dinutuximab (Unituxin^®^) is Food and Drug Administration (FDA)-approved as part of post-consolidation therapy for pediatric high-risk neuroblastoma, while dinutuximab beta (Qarziba^®^) is authorized in Europe for high-risk and selected relapsed/refractory disease. In the pivotal COG ANBL0032 trial, addition of dinutuximab plus GM-CSF and IL-2 to isotretinoin maintenance after induction and consolidation therapy improved 2-year event-free survival from 46% to 66% and overall survival from 75% to 86% in eligible patients, establishing anti-GD2 immunotherapy as the standard of care in this setting [109].

Naxitamab (Danyelza^®^) is FDA-approved with GM-CSF for relapsed or refractory high-risk neuroblastoma involving bone or bone marrow [110], extending anti-GD2 therapy into the relapsed/refractory setting. Its accelerated FDA approval in 2020 was supported by single-arm clinical data, and subsequent results have further characterized objective response rates of 40–50% [111].

Anti-GD2 efficacy depends on antigen density and immune effector function, while treatment is limited by on-target neuropathic pain and other toxicities, including capillary leak, hypotension, and hypersensitivity [112,113]. Developmental plasticity creates an additional vulnerability, as a transition toward the MES state can suppress ST8SIA1, lower GD2, and confer resistance [56]. Additionally, YAP can reinforce ST8SIA1/GD2 suppression, and human tumors can contain spatially intermixed GD2-high and GD2-low/negative populations [57,66]. Epigenetic or signaling-based restoration of ST8SIA1/GD2 therefore represents a potential strategy for resensitizing antigen-low tumors, although clinical validation is still required.

### 7.2. Emerging Therapies

Most approaches that aim to manipulate the sialome remain on the preclinical or early clinical level and fall into two categories. One strategy reduces or removes tumor sialylation using sialyltransferase inhibitors, glycomimetics, or targeted sialidases. Another strategy blocks inhibitory Siglec signaling without globally desialylating tissues [114,115]. Small-molecule inhibition of the polysialyltransferase ST8SIA2 reduces polySia-NCAM expression and tumor cell migration in vitro, and nanoparticle-based delivery of the transition-state sialyltransferase inhibitor analogs is being explored to improve their pharmacologic stability and tumor targeting [97,114]. Rationally designed glycomimetics such as P-3Fax-Neu5Ac achieve a related endpoint chemically, competitively blocking sialic acid biosynthesis. Targeted nanoparticle delivery of this compound suppresses metastatic spread in preclinical melanoma models [116]. Nanomedicine platforms more broadly are being adapted to co-deliver glycan-targeting payloads with immunomodulatory agents [117], extending the logic of targeted sialic acid blockade beyond single-agent therapy. Sialidase-based strategies disrupt inhibitory Siglec-5 and Siglec-10 signaling and convert immunologically “cold” tumors into “hot” ones, potentiating chimeric antigen-receptor–macrophage activity in preclinical models [118]. An engineered sialidase-Fc fusion protein built on the same antibody–sialidase conjugate principle has undergone phase 1/2 clinical evaluation, alone and in combination with anti-PD-1 blockade, across multiple solid tumor types (GLIMMER-01, NCT05259696), providing clinical proof of pharmacodynamic desialylation and immune activation [119,120,121,122]. Direct Siglec blockade with antagonist antibodies is being pursued as a complementary means of reversing this glyco-immune checkpoint [115]. These approaches are conceptually attractive for neural-crest-derived tumors, but their efficacy and therapeutic window in pediatric disease remain unresolved. There are also specific safety considerations. Because peripheral nerves contain GD2 and can exhibit developmental or injury-regulated polysialylation, systemic or incompletely tumor-selective desialylation could also alter glycans on healthy neural tissues. It is not yet known whether this would alleviate toxicity by removing the antigen recognized by anti-GD2 antibodies or worsen it by generating neo-epitopes or otherwise perturbing the nerve glycocalyx. Antigen-selective approaches such as antibodies directed against the tumor-enriched O-acetylated GD2 derivative, which is mostly absent from peripheral nerves, are one strategy for reducing nerve cross-reactivity [123].

Manipulating sialylation can also be therapeutically useful in the opposite direction by increasing a defined sialylated antigen rather than globally reducing the sialome. In neuroblastoma models, a cell-permeable sialic acid analog increased intracellular CMP-Neu5Ac and enhanced GD2 expression, while HDAC inhibition increased ST3GAL5 and ST8SIA1 expression. Combining these two approaches further increased surface GD2 [124]. Pathogenic sialylation may be reduced when it supports immune evasion, whereas a therapeutically useful sialoglycan such as GD2 may instead be restored or increased to improve antigen-directed treatment. Whether such antigen-boosting strategies can be translated safely into humans remains unresolved. This antigen-boosting logic carries the opposite safety concern compared to the sialidase strategies discussed above. Increasing GD2 synthesis could plausibly raise GD2 density on peripheral nerves as well as on tumor cells and thereby exacerbate the complement-mediated allodynia that already limits anti-GD2 therapy (Section 5.1). Whether GD2-boosting agents can be restricted to the tumor cells or combined with strategies to spare peripheral-nerve GD2 has not been tested.

Cellular immunotherapy extends antigen targeting beyond monoclonal antibodies. In the final analysis of a phase 1/2 trial of GD2-CART01 in children with high-risk metastatic, relapsed, or refractory neuroblastoma, the overall response rate was 66%, with a 5-year overall survival of 42.7% [125]. Outcomes were more favorable in the target population, defined as patients treated with low disease burden at the recommended dose of CAR-positive cells, with an overall response rate of 77% and 5-year overall survival of 68%. These findings demonstrate the potential for durable responses with GD2-directed cellular therapy, particularly when administered at a low disease burden.

### 7.3. Biomarkers and Precision Medicine

**Figure 4:**
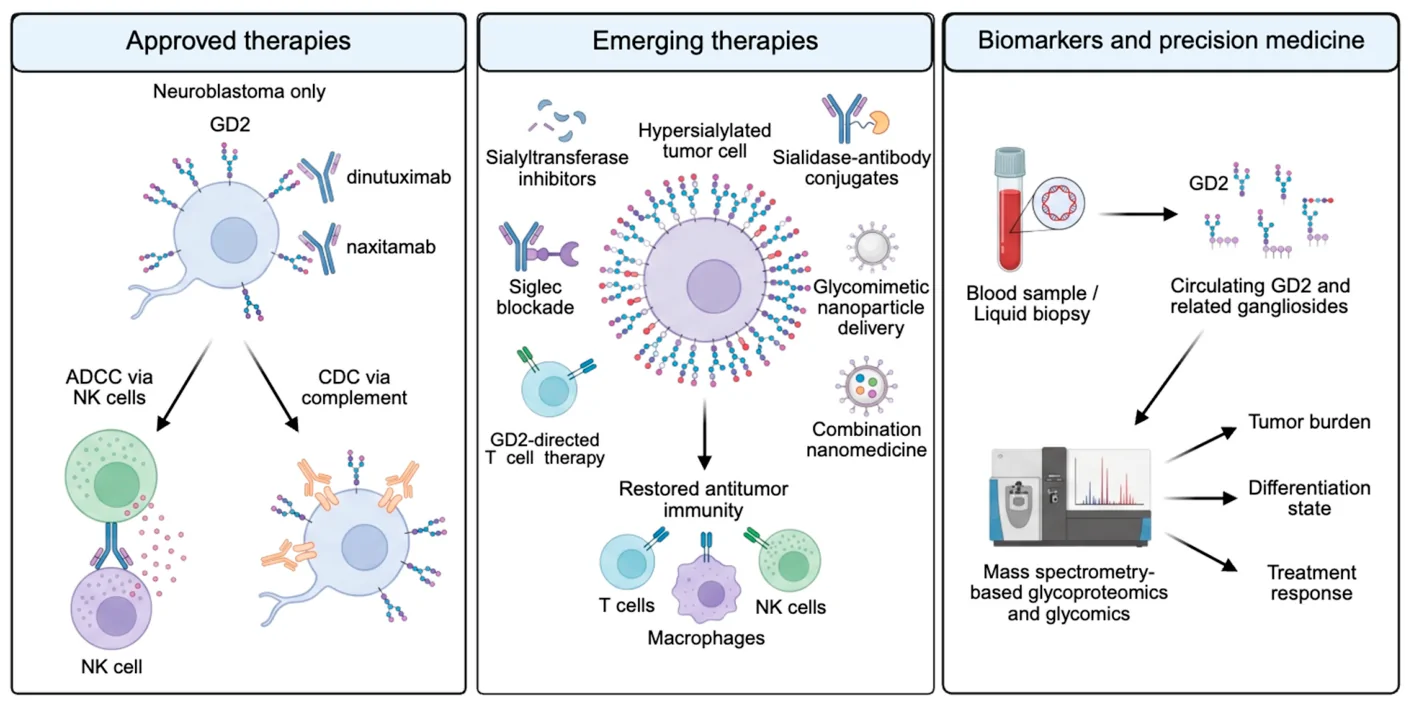
Therapeutic strategies targeting sialic acid biology in neuroblastoma and other neural-crest-derived pediatric tumors, spanning approved, emerging, and precision-medicine approaches. (**Left**) Approved therapies. Approved GD2-targeted monoclonal antibodies, including dinutuximab and naxitamab, bind GD2 ganglioside on the tumor cell surface and mediate tumor cell killing through two mechanisms: antibody-dependent cellular cytotoxicity (ADCC), in which NK cells recognize antibody-coated tumor cells and release cytotoxic granules, and complement-dependent cytotoxicity (CDC), in which complement activation directly lyses the tumor cell. (**Middle**) Emerging therapies. Six strategies in preclinical or early clinical development, conceptually applicable across neural-crest-derived tumors: sialyltransferase inhibitors that reduce tumor sialylation; sialidase–antibody conjugates that desialylate tumor cells; Siglec blockade that relieves inhibitory signaling on T cells, macrophages, and NK cells; glycomimetics delivered in nanoparticle formulations; GD2-directed T cell therapy (including CAR T cells); and combination nanomedicine platforms. Collectively, these approaches seek to enhance antitumor immune activity through glycan-directed or glycan-dependent mechanisms. (**Right**) Biomarkers and precision medicine. Circulating ganglioside measurements, including GD2, are being explored as minimally invasive markers of tumor burden, differentiation state, and treatment response in neuroblastoma, with related detection approaches being investigated across other pediatric tumors. Mass spectrometry-based glycoproteomics and glycomics add protein-, site-, and structure-level detail that complements these circulating biomarkers, moving toward panels for diagnosis, risk stratification, and monitoring of treatment response. Figure created in BioRender. Stafman. (2026) https://app.biorender.com.

Biomarker development is proceeding in parallel with more precise deployment of these therapies. Circulating GD2-related ganglioside measurements are being explored as minimally invasive markers of tumor burden, differentiation state, and response [126]. Mass spectrometry-based glycoproteomics and glycomics can add protein-, site-, and structure-level information that is inaccessible from transcriptomic measurements alone [127]. Together, these approaches (Figure 4) are promising, but most remain investigational and should not yet be equated with established clinical risk stratification.

## 8. Emerging Technologies/Future Directions

Several unresolved questions will determine whether developmental sialylation becomes a useful framework for these tumors. Cell-state-resolved maps are needed to establish which glycans track developmental identity. Functional studies must distinguish markers from drivers. Longitudinal sampling is required to determine how treatment and relapse alter the sialome. Any therapeutic target will ultimately require sufficient selectivity over normal developing tissue.

### 8.1. Multi-Omic Approaches

Because glycan structures are not directly templated by the genome, single-cell glycomics requires specialized analytical strategies. DNA-barcoded lectins can convert glycan-binding events into sequenceable readouts, while high-sensitivity capillary-electrophoresis–mass-spectrometry workflows can directly profile glycans from very small cell populations [128]. Spatial derivatization and mass spectrometry imaging can also distinguish α2,3- from α2,6-linked sialic acids within tissue sections [129]. The clinical value of this spatial resolution is illustrated in ovarian cancer, where mass spectrometry imaging showed that specific N-glycan families preferentially localize to tumor regions in late-stage disease [130]. This stage-specific pattern would be obscured entirely in bulk glycomic analysis. Applying these tools to neuroblastoma and other neural-crest-derived tumors could determine whether ADRN, MES, stromal, and immune compartments occupy distinct sialylation states rather than averaging them together in bulk measurements.

The single-cell and spatial multi-omics infrastructure into which these glycan-specific tools are being integrated is expanding rapidly, with a growing number of platforms accessible through open community resources [131,132]. This infrastructure is already reshaping neuroblastoma biology. Single-cell RNA sequencing has resolved a shared pan-neuroblastoma tumor cell identity across patients [133] and has traced its cell of origin to specific sympathoblast lineages in the developing human adrenal medulla [134]. Integrating glycomic readouts with these established cell state maps would allow the central hypothesis of this review to be tested at cellular resolution, specifically whether tumor-associated sialylation tracks with developmental lineage, therapy-induced state transitions or both. A decisive test of the developmental-sialylation model will require paired reference maps spanning normal fetal neural-crest-derived lineages, treatment-naïve tumors, defined ADRN and MES states, and post-treatment or relapsed disease, followed by functional perturbation of candidate glycan regulators.

Glycoproteomics adds another essential dimension by identifying the proteins and sites that carry specific glycans [135]. In neuroblastoma, serum N-glycan profiling has identified a six-glycan signature that distinguishes neuroblastoma patients from controls with accuracy [136]. Site-specific methods have also expanded the ability to measure polysialylation, a modification carried by a limited set of proteins, including NCAM and neuropilin-2, rather than being broadly distributed across the glycoproteome [137]. Separately, metabolic engineering of cell-surface sialylation with synthetic sugar analogs selectively reduces neuroblastoma cell migration and invasion [138]. Combining mass spectrometry imaging with glycoproteomic analysis has also begun to link specific dysregulated sialylated glycans directly to their carrier proteins within tumor tissue, as shown for haptoglobin in canine glioma [139]. A similar strategy could be applied to neural-crest-derived tumors. Along with liquid biopsy-based glycoproteomic profiling [127], these methods could link a structural glycan change to its carrier protein and ultimately to the phenotype, which is the level of evidence needed to distinguish biomarkers from mechanisms.

### 8.2. Functional and Developmental Models

CRISPR-based functional genomics is beginning to map the genetic control of cancer-associated sialoglycans. Genome-wide CRISPR activation screens have shown that Siglec-ligand display is shaped by competition among glycosyltransferase pathways and by expression of specific glycoprotein ligands that present sialylated glycans at the cell surface [140]. A lectin-based magnetic CRISPR screening approach has been developed to identify regulators of cell surface sialylation [141], and curated tools such as the GlycoGene CRISPR library facilitate systematic loss-of-function studies across glycosylation pathways [142]. Related CRISPR screens reinforce the principle that cell surface glycan phenotypes emerge from coordinated Golgi processing networks rather than from any single sialyltransferase [143]. Applying these tools directly to pediatric neural-crest-derived tumor models may help distinguish causal glycan regulators from correlative markers.

Because human and mouse neural crest development diverge in timing and gene regulation, mouse models capture only part of the biology relevant to human pediatric tumors. Human pluripotent stem-cell-derived neural crest models profiled by single-cell sequencing have shown that neuroblastoma-associated chromosomal gains (17q, 1q) and increased MYCN activity disrupt the same developmental gene networks that are dysregulated in neuroblastoma tumors, directly linking the developmental arrest model to a tractable experimental system [144]. A complementary human-induced pluripotent-stem-cell-derived three-dimensional model has recently been reported to generate neuroblastoma organoids, offering a platform in which tumor initiation can be observed alongside normal neural crest differentiation [145]. These organoid systems belong to a broader family of pluripotent-stem-cell-derived brain and neural crest models that allow developmental and tumorigenic trajectories to be compared side by side [146], while patient-derived xenograft and cell line models remain essential given the rarity of neuroblastoma [147,148]. Layering glycomic profiling and functional perturbation onto these developmentally faithful models allows direct, mechanistic testing of whether tumor-associated sialylation represents retention of developmental state, reacquisition after lineage state change, de novo remodeling during malignancy, or combinations of these processes.

### 8.3. Clinical Translation

Sialylated glycan biomarkers already show substantial translational potential in neuroblastoma. In a retrospective cohort, circulating GD2 was 30-fold higher in children with high-risk neuroblastoma at diagnosis than in healthy controls, supporting its potential as a circulating tumor biomarker [149]. Subsequent studies have confirmed that specific circulating GD2 lipoforms are elevated in neuroblastoma patients [150]. Complementary liquid biopsy approaches are also emerging. Flow cytometric detection of GD2 taken up by circulating monocytes, rather than free GD2, has shown high sensitivity in neuroblastoma and is now being explored across other GD2-positive pediatric tumors, including those of the central nervous system [151]. The serum N-glycan panel described above also provides an independent, sialylation-specific readout that separates neuroblastoma patients from controls [136], and broader advances in AI-assisted glycoproteomics are expanding the number of cancer- and organ-specific glycosylation signatures with diagnostic and prognostic potential.

Realizing this potential for precision medicine requires connecting biomarker discovery back to developmental validation. Because tumor and developmental sialylation programs overlap, glycomic biomarkers and the neuroblastoma organoid and stem cell models described above will need reciprocal validation before either can be deployed with confidence. GD2-based liquid biopsy assays are being investigated for diagnosis, disease monitoring, and detection of relapse, with the potential to complement established clinical and molecular markers [151]. A critical next step is longitudinal sampling before, during, and after therapy to determine whether circulating or tissue glycan changes reflect tumor burden, selection of antigen-low states, or true lineage transitions. Ultimately, integrating glycomic measurements with functional perturbation and developmental models may determine whether sialylation adds clinically useful information beyond histology and tumor genomics.

## 9. Conclusions

Current evidence does not support a single conserved “neural crest sialylation program” across neural-crest-derived tumors. Instead, specific sialoglycan pathways appear to be retained, reacquired, or independently remodeled in a tumor- and cell-state-dependent manner. Neuroblastoma provides the strongest paradigm. Developmental ganglioside biology generates the clinically actionable antigen GD2, polySia-NCAM supports migratory behavior, and ADRN-MES cell state transitions alter the glycosyltransferase machinery that determines surface antigen density and therapeutic sensitivity.

Much of the available evidence remains associative (Table 1). Direct mechanistic evidence linking specific sialylation changes to neural crest induction, lineage commitment, tumor initiation or bona fide reuse of a developmental glycan state remains limited, particularly outside neuroblastoma and melanoma. Even in melanoma, strong cancer-glycobiology mechanisms should not automatically be interpreted as evidence of developmental neural crest reuse. MPNST, pheochromocytoma/paraganglioma, and schwannoma therefore represent major opportunities for comparative studies that pair developmental models with tumor glycomics and functional perturbation.

The pediatric-developmental perspective also changes how tumor-associated sialylation should be interpreted. For a childhood tumor arising from an incompletely mature neural crest lineage, a tumor-associated glycan may represent retention of a developmental surface state rather than re-expression after years of normal differentiation. Conversely, therapy-induced state transitions may permit reacquisition of a previously lost developmental program, whereas other changes may reflect de novo remodeling unique to malignancy. Longitudinal, cell-state-resolved studies that include matched developmental references will be required to distinguish these possibilities and to determine which provides the greatest tumor-selective therapeutic window.

Clinically, GD2-directed therapy demonstrates that a developmentally enriched and relatively restricted glycan can become an actionable tumor antigen. Whether polysialylation, other sialyltransferases, gangliosides, or sialoglycan–Siglec interactions offer comparable therapeutic selectivity remains uncertain. Resolving that question will require the mapping of developmental states, longitudinal tumor sampling, and functional experiments that distinguish causal glycan changes from biomarkers of cell state.

## Data Availability

No new data were created or analyzed in this study. Data sharing is not applicable to this article.
